# Overlapping activation pattern of bitter taste receptors affect sensory adaptation and food perception

**DOI:** 10.3389/fnut.2022.1082698

**Published:** 2022-12-19

**Authors:** Roman Lang, Tatjana Lang, Andreas Dunkel, Florian Ziegler, Maik Behrens

**Affiliations:** Leibniz Institute for Food Systems Biology at the Technical University of Munich, Freising, Germany

**Keywords:** bitter taste receptor, calcium mobilization assay, sensory adaptation, TAS2R, chicory, coffee (*C. arabica*)

## Abstract

The composition of menus and the sequence of foodstuffs consumed during a meal underlies elaborate rules. However, the molecular foundations for the observed taste- and pleasure-raising effects of complex menus are obscure. The molecular identification and characterization of taste receptors can help to gain insight into the complex interrelationships of food items and beverages during meals. In our study, we quantified important bitter compounds in chicory and chicory-based surrogate coffee and used them to identify responsive bitter taste receptors. The two receptors, TAS2R43 and TAS2R46, are exquisitely sensitive to lactucin, lactucopicrin, and 11β,13-dihydrolactucin. Sensory testing demonstrated a profound influence of the sequence of consumption of chicory, surrogate coffee, and roasted coffee on the perceived bitterness by human volunteers. These findings pave the way for a molecular understanding of some of the mixture effects underlying empirical meal compositions.

## Introduction

Of the five basic taste qualities sour, salty, sweet, umami, and bitter, bitter taste is devoted to detect potentially harmful food components (1). However, there is no strict correlation between bitterness and toxicity (2) and many bitter substances are components of edible vegetables, and may even have health-beneficial effects or serve as medicine (3). The recognition of the, at least, hundreds of chemically diverse bitter compounds (4) relies in human on ∼25 taste 2 receptor genes (*TAS2R*), which are expressed in a heterogeneous pattern in taste buds of the oral cavity (5). Over the past years, 21 of the 25 TAS2R have been de-orphaned revealing grossly differing tuning-breadths and, depending on the bitter agonists, detection ranges from high nanomolar to low millimolar concentrations (6, 7). In many cases, the concentration ranges resulting in the activation of heterologously expressed TAS2R have been shown to correlate well with human bitter taste sensitivities for individual compounds making these assays highly valuable tools to investigate human bitter taste (8–10). A very rich source of bitter compounds is coffee. Although not all coffee constituents known to taste bitter have been analyzed for their TAS2R activation profile until now (11, 12), some prominent bitter substances were tested. These tests revealed that the perhaps most well-known bitter compound in coffee, caffeine, activated in total five TAS2Rs, namely TAS2R7, −R10, −R14, −R43, and −R46 (6), whereas a whole array of additional substances, mozambioside, bengalensol, kahweol, and cafestol activate TAS2R43 and TAS2R46 and hence, a subset of the caffeine-responsive receptors (13).

Although evidence for health beneficial effects of moderate consumption of roasted coffee is mounting [for a review see Sirotkin and Kolesárová (14)], adverse effects such as gastroesophageal reflux symptoms caused by coffee’s acidity and caffeine (15) and sleeplessness as a consequence of the pharmacological activity of caffeine [for a review see Wikoff et al. (16)], represent reasons for some consumers for choosing surrogate coffee instead of roasted coffee. One of such surrogate coffees frequently consumed contains, with or without the addition of other plant materials, ground roasted chicory roots, which contribute to flavor similarities with roasted coffee (17). Today, these products are still on the market, mostly consumed by health-conscious consumers or children. Chicory (*Cichorium intybus L.*) contains a variety of sesquiterpene lactones of germacranolide and guajanolide types, which were known for their bitter taste already in the 19th century [for a review see Janda et al. (18)]. As the purpose for the generation of these surrogate coffees has been to match the taste of roasted coffee including its bitterness, we wondered why in particular chicory was chosen as the dominant constituent and whether similar TAS2R-profiles are activated by coffee and chicory bitter substances.

In the current study, we used a heterologous expression assay to screen all 25 human TAS2Rs with lactucopicrin, a bitter compound from chicory, to assess their activation profile. The responsive receptors were further tested with lactucopicrin and two related compounds from chicory to determine the concentration ranges at which they induce TAS2R responses. The obtained activation profiles were compared with those established for roasted coffee to investigate the similarities regarding bitterness. The corresponding substances were quantified in chicory and chicory-based surrogate coffee and the resulting data compared with their *in vitro* determined effective concentration ranges. By sensory experiments, the bitter taste impressions of human volunteers were recorded to test if cross-adaptation arises from the sequential stimulation with chicory/surrogate coffee and roasted coffee.

## Materials and methods

### Chemicals

Lactucin [purity > 95% (HPLC)], lactucopicrin [purity > 90% (HPLC)], and 11β,13-dihydrolactucin [purity > 95% (HPLC)] were purchased from Extrasynthèse S.A. (Genay, France). Other compounds were obtained from Sigma-Aldrich (Steinheim, Germany). The bitter compounds were dissolved as stock solution (10 mM) in dimethyl sulfoxide (DMSO) and stored at −20°C.

### Ultra high performance liquid chromatography

The chromatographic system consisted of a Shimadzu Nexera X2 ultra high performance liquid chromatography (UHPLC) system (Shimadzu, Duisburg, Germany), comprising of an autosampler (SIL 30AC, kept at 15°C), two pumps (2 × LC-30AD), a degasser (DGU 20 A5R), a column oven (CTO 30A, kept at 40°C), and a system controller (CBM 20A). The UHPLC system was connected to an AB Sciex 5500 Qtrap mass spectrometer (Sciex, Darmstadt, Germany) operating in positive electrospray mode. Analyst 1.6.2 was used for instrument control and data analysis. The settings were as follows: Curtain Gas, 40; collision gas, “medium”; ion spray voltage, + 5.5 kV; source temperature, 550°C; nebulizer gas, 60; and heater gas, 50. Resolution was set to “unit.” The dwell time for each mass transition was 20 ms with 5 ms between mass transitions. Two MRM-traces per compound were recorded (DP, EP, CE, CXP, quantifier is marked with *): Lactucin 276.97 > 213.0*/114.9 (161,10,17,4/161,10,77,6); lactucopicrin 411.04 > 215.0*/114.9 (86,10,17,4/86,10,77,6); 11β,13-dihydrolactucin 278.99 > 215.0*/159.0 (151,10,17,12/151, 10,27,10). The samples were separated on a Kinetex C18 column (1.7 μm, 100 mm × 2.1 mm, Phenomenex, Aschaffenburg, Germany) with 0.1% formic acid in water (eluent A) and 0.1% formic acid in acetonitrile (eluent B) at a flow rate of 400 μl/min. After injection (1 μl), eluent B was increased from 5 to 95% in 5 min with a non-linear gradient (curve 5) followed by 2 min of isocratic elution. The starting conditions were re-established within 0.5 min, and equilibration was 2.5 min prior to the next injection. Four minutes after sample injection, the ECHO standard (1 μM, 1 μl) was injected.

### Quantification of lactucin, lactucopicrin, and 11β,13-dihydrolactucin

#### Standards

Individual stock solutions of the analytes lactucin, lactucopicrin, and 11β,13-dihydrolactucin were prepared at 1 mM in DMSO/methanol 1/9. Aliquots (100 μl) of these stocks were combined and diluted with ACN to 10 μM, and further diluted with ACN in 1 + 1 steps to obtain standards for calibration at 10, 5, 2.5, 1.25, 0.625, 0.313, 0.156, 0.078, 0.039, and 0.019 μM.

We used lactucin as the pseudo-internal ECHO standard for normalization of the analyte peak areas (19, 20). An aliquot of the lactucin stock (1 mM in DMSO/methanol 1/9, v/v) was diluted with ACN to a final concentration of 1 μM.

Calibration standards were analyzed in triplicates and area ratios (analyte/ECHO standard) plotted vs. concentration ratios (analyte/ECHO standard) with 1/× weighing (see Supplementary Table 1). Calibration curves (linear regression) were lactucin/ECHO lactucin *y* = 1.50× + 0.006 (*R*^2^ = 0.998), lactucopicrin/ECHO lactucin *y* = 17.5× + 0.106 (*R*^2^ = 0.995), 11β,13-dihydrolactucin/ECHO lactucin *y* = 2.79× + 0.008 (*R*^2^ = 0.999). Quantitative data were calculated from the peak ratios of analyte/ECHO standard and the respective calibration curve.

#### Sample preparation

The homogenized sample (1–4 g) was weighed into a measured flask (100 ml), suspended in 10% aqueous acetonitrile and incubated with ultrasonication (30 min, 40°C). The flask was filled to the mark with 10% acetonitrile after cooling to room temperature. After membrane filtration, an aliquot (1 μl) was injected into the UPLC-MS/MS system. Liquid samples (coffee brew and beverage prepared from roasted chicory root) were directly injected (1 μl). Samples were kept frozen until analysis (−20°C) and exposure to light was avoided (21).

Quality control (QC) samples were prepared in aqueous solution (10% ACN) and analyte-free matrix (coffee brew) by addition of 1 μM of the analytes. Precision, accuracy, lower limit of detection (LloQ) and linear range are given in Supplementary Table 2 based on QC samples and calibration standards (22).

### Functional calcium mobilization assay

#### Screening

As described previously (13, 23), HEK 293T-Gα16gust44 cells were cultivated on poly-D-lysine coated 96-well-plates under standard conditions [Dulbecco’s Modified Eagle Medium (DMEM), 10% FCS, 1% penicillin/streptomycin, 1% glutamine; 37°C, 5% CO_2_, saturated air humidity] and transiently transfected with expression constructs coding for the 25 TAS2Rs using lipofectamine 2000 (Thermo Fisher Scientific, Darmstadt, Germany). A transfection with empty expression vector was included for a negative control (mock). Duplicate wells were prepared for each compound-receptor combination. After transfection (∼24 h), cells were loaded with calcium-sensitive dye (Fluo4-AM, Thermo Fisher Scientific, Darmstadt, Germany) in the presence of probenecid (2.5 mM, Sigma-Aldrich, Steinheim, Germany) for 1 h, washed with C1 buffer (130 mM NaCl, 5 mM KCl, 2 mM CaCl_2_, 10 mM glucose, 10 mM HEPES; pH 7.4), stored in the dark for 30 min and finally washed once more. Cells were then placed in a fluorometric imaging plate reader (FLIPR*^Tetra^*, Molecular Devices, San Jose, CA, USA). The bitter compounds, dissolved in the C1 buffer, were automatically administered to the cells and changes in fluorescence were monitored. For screening, we used 1 and 10 μM of lactucopicrin. Vitality of cells was evaluated by subsequent addition of somatostatin 14 (100 nM, Bachem, Bubendorf, Switzerland).

#### Dose-response relationships

The stable inducible cell lines FLP-In T-REX 293-Gα16gust44-TAS2R14, -TAS2R43 were available from previous research (24), the corresponding TAS2R46 expressing cell line was generated accordingly (24), by simultaneous transfection of FLP-In T-REX 293-Gα16gust44 cells with cDNA of TAS2R46 in pcDNA5/FRT/TO vector and the FLP-recombinase encoding plasmid pOG44 using lipofectamine 2000. Subsequent treatment with 100 μg/ml hygromycin B selected cells with successful integration. Cells were cultivated under the same conditions as in the screening until a confluence of ∼70% was reached. Next, cells were treated with 5 μg/mL tetracycline to induce receptor expression. Non-induced cells served as negative control (mock). Induction time for TAS2R14 and TAS2R43 was 14–18 h, and for TAS2R46 3–5 h. For the experiment, bitter compounds were dissolved in the C1 buffer (final concentrations between 0.01 and 100 μM). As a positive control for TAS2R14 and TAS2R43 aristolochic acid (Sigma-Aldrich, Steinheim, Germany) and for TAS2R46 strychnine (Sigma-Aldrich, Steinheim, Germany), respectively, was applied. All other steps were identical to the screening.

#### Determination of threshold concentrations and half-maximal effective concentrations (EC_50_) of TAS2R14, TAS2R43, and TAS2R46

The assessment of data was based on three independent experiments each performed in duplicates. For calculation of ΔF/F, the fluorescence changes of non-induced cells were subtracted from the corresponding measurements of tetracycline-induced cells. The resulting signals were normalized to background fluorescence. The signal amplitudes were plotted vs. the log concentrations of the compounds to obtain individual dose-response-curves. The half-maximal effective concentrations (EC_50_) were calculated with SigmaPlot (v14.0) by non-linear regression using the equation *y* = *(max–min)/[1* + *(x/EC_50_)*^–Hillslope^*]* + *min*.

### Human sensory study

#### Study participants

Individuals volunteering to participate in the human sensory study were recruited at the Leibniz Institute for Food Systems Biology at the Technical University Munich. The line-up consisted of 16 individuals (7 women, age 36 ± 12 years; 9 men, age 37 ± 10 years). Sensory experiments were done in the morning (10:00–12:00) and early afternoon (14:00–15:00) in four sessions. Volunteers were asked to refrain from food and drinks other than tap water 30 min prior to sensory evaluation. In every experiment, food item 1 (1 ml or 1 g, respectively) was taken into the mouth and bitterness was evaluated. The samples were moved in the mouth for a total time of 30 s and swallowed or expectorated and the oral cavity was rinsed with water (5 ml). Immediately after rinsing, food item 2 (1 ml or 1 g, respectively) was evaluated. There were 30 min between the sensory sessions. The study protocol was approved by the ethical committee of the faculty of medicine of the Technical University Munich (2022-203-S-NP). Written informed consent was obtained from the participants.

#### Evaluation of bitterness

Bitterness was evaluated on a labeled magnitude scale (see Supplementary Figure 1). After the test the labels were translated into numbers from 0–100 for quantification: “no bitterness” = 0, “hardly perceived” = 1.5, “weak” = 6, “moderate” = 17, “strong” = 35, “very strong” = 52, and “strongest imaginable perception” = 100 [modified from (25)].

#### Foods

The administered coffee brew was freshly prepared as follows: coffee powder (48.75 g, 100% Arabica, Tchibo GmbH, Hamburg, Germany) were weighed into a French press, mixed with freshly boiled table water (Evian^®^, 750 ml) and incubated (4 min). The press was passed through the suspension and the clear coffee brew was poured. Following the recommendations of the manufacturer (Naturata, Finzel and Schuck GmbH, Limbach-Oberfrohna, Germany), brew from coffee surrogate (100% roasted chicory root) was prepared similarly using 30 g of powder and 750 ml of boiling water. Fresh chicory was obtained from local stores and used immediately for sensory experiments after homogenization. Bottled water (Evian^®^) served for rinsing the oral cavity.

### Statistical analysis

Determination of threshold concentrations in functional receptor experiments.

The assessment of functional receptor data was based on three independent experiments each performed in duplicates. Determinations of threshold concentrations were done with MS Excel using Student’s *T*-test with *p* < 0.01 considered as significant.

#### Statistical analysis of taste tests

Statistical analysis of taste tests was done by One-way repeated measures ANOVA with Bonferroni’s *t*-test using SigmaPlot (14.0) with *p* < 0.02 considered as significant.

## Results

### Identification of TAS2Rs responding to chicory bitter substances

**FIGURE 1:**
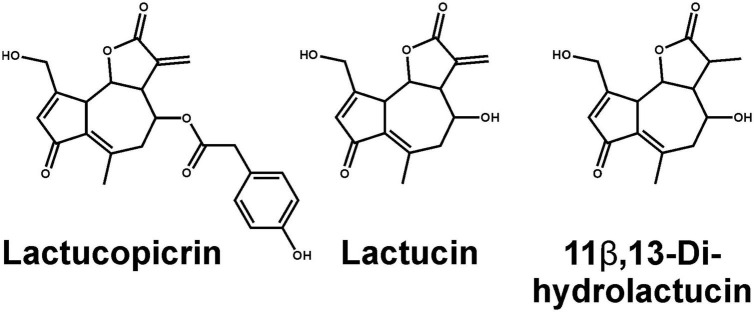
Chemical structures of main bitter compounds from chicory. Shown are lactucopicrin used for TAS2R screening **(left)**, lactucin **(middle)**, and a naturally occurring hydrogenated form of lactucin, 11β,13-dihydrolactucin **(right)**.

**FIGURE 2:**
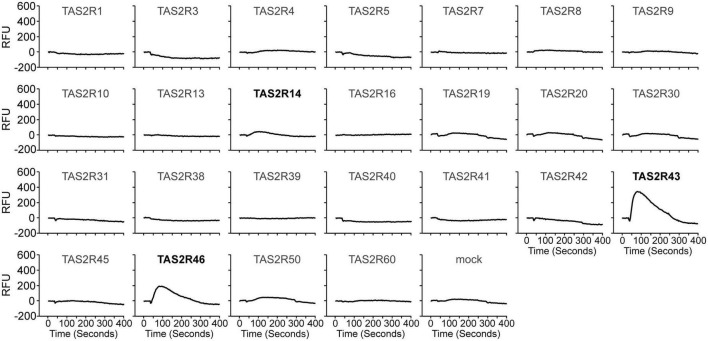
Human bitter taste receptors responding to lactucopicrin. Fluorescence traces of HEK 293T-Gα16gust44 cells upon stimulation with 10 μM lactucopicrin are shown. Lactucopicrin-activated receptors are printed bold and in black. As negative controls, cells were transfected with empty vector (mock). *Y*-axis, relative fluorescence units (RFU); *x*-axis, time in seconds.

Historically, roasted coffee has sometimes been replaced by apparently similar tasting surrogates made from ground roasted chicory roots containing a variety of bitter sesquiterpene lactones. Why in particular chicory roots were chosen and if, for example, bitter compounds from roasted coffee and chicory root exhibit similar TAS2R activation profiles is not established. To identify TAS2Rs responding to chicory-based surrogate coffee and fresh chicory, we screened the substance lactucopicrin (Figure 1) known to contribute to the bitter taste of chicory (18) for the activation of 25 human TAS2Rs. We observed responses with cells expressing TAS2R14, TAS2R43, and TAS2R46 (Figure 2). Whereas TAS2R14 showed only small changes in fluorescence, the other two TAS2Rs exhibited pronounced signals.

### Determination of dose-response relationships for identified receptor-agonist combinations

**FIGURE 3:**
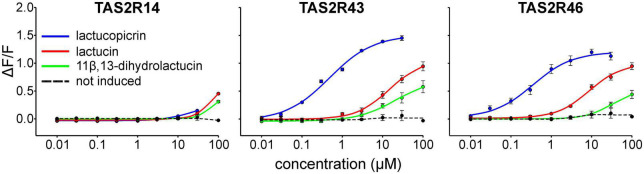
Dose-response relationships of TAS2Rs activated by chicory bitter substances. Three cell lines stably expressing the chimeric G protein Gα16gust44 as well as receptors TAS2R14, TAS2R43, or TAS2R46 (tetracycline-inducible) were subjected to calcium mobilization assays. Administration of lactucopicrin (blue curves), lactucin (red curves) and 11β, 13-dihydrolactucin (green curves) and measurement of fluorescence changes were done using a fluorometric imaging plate reader (FLIPR^tetra^). For negative controls, non-induced cells were challenged with the test compounds and responses shown as averaged curve (dotted, black). Relative changes of fluorescence (ΔF/F) are plotted on the *y*-axis. Concentrations of applied test compounds in μM are indicated at the logarithmically scaled *x*-axes.

To assess the effective concentration ranges of lactucopicrin and the related bitter substances found in chicory, lactucin and 11β,13-dihydrolactucin (Figure 1), we determined their corresponding dose-response relationships using stable inducible cell lines expressing TAS2R14, −R43, and −R46 (Figure 3). Whereas the three compounds resulted only in small responses of TAS2R14 expressing cells at the highest concentrations, TAS2R43 and TAS2R46 were potently and effectively activated (Figure 3). All three substances showed the same rank-order-of-potency at both receptors, with lactucopicrin exhibiting the highest potency, lactucin with medium potency and 11β,13-dihydrolactucin being least potent. The TAS2R46 was more selective between lactucin and 11β,13-dihydrolactucin as evident from the pronounced shift in the dose-response relationships. The determined threshold and EC_50_-concentrations are provided in Table 1.

**TABLE 1:** Threshold- and EC_50_-concentrations of test compounds.

Compound	TAS2R14		TAS2R43		TAS2R46	
	Threshold	EC_50_-conc.	Threshold	EC_50_-conc.	Threshold	EC_50_-conc.
Lactucopicrin	30 μM	n.d.	0.1 μM	0.46 ± 0.08 μM	0.03 μM	0.35 ± 0.06 μM
Lactucin	100 μM	n.d.	10 μM	14.9 ± 2.5 μM	1 μM	8.85 ± 1.17 μM
11β,13-Dihydrolact	100 μM	n.d.	10 μM	n.d.	30 μM	n.d.

The threshold concentrations were determined as the lowest concentrations leading to statistically significant differences from the corresponding non-induced signals (Student’s *T*-test, *p* < 0.01). N.d., not detected.

### Determination of chicory bitter compound concentrations in food and beverages

Next, we determined the concentrations of the three compounds in fresh vegetables, roasted coffee and surrogate coffee as well as in brews made from roasted coffee and surrogate coffee, to test if the concentrations present in these samples match the bitter receptor activating concentrations determined *in vitro*. The data clearly demonstrate that chicory, radicchio, and surrogate coffee powder contain concentrations of the compounds that exceed receptor activating concentrations (Table 2). For example surrogate coffee powder contained 32.48 μmol/g lactucin, which exceeds the EC_50_-concentration found in the receptor assays by more than 1000-fold. The same is true for lactucopicrin (10.66 μmol/g) and, when extrapolating the dose-response relationships, also for 11β,13-dihydrolactucin (52.69 μmol/g). Chicory contains 42.13 μmol/g lactucin, 11.93 μmol/g of lactucopicrin and 6.18 μmol/g 11β,13-dihydrolactucin, and thus should allow for maximal activation of the corresponding TAS2Rs. We further found that these compounds are, as anticipated, absent from roasted coffee, whereas surrogate coffee brew contained 1.35 μM lactucin, 0.45 μM lactucopicrin, and 1.98 μM 11β,13-dihydrolactucin. As the 11β,13-dihydrolactucin concentration found in surrogate coffee is below the threshold concentrations determined for the three receptors, we would exclude this compound as being responsible for the bitterness of surrogate coffee brew. Lactucin just reaches the threshold concentration of TAS2R46, whereas the lactucopicrin concentration is approximately equal to the EC_50_-concentration of this substance at the TAS2R43 and exceeds the EC_50_-concentration determined for the TAS2R46. Thus, the bitterness of surrogate coffee brew should be dominated by lactucopicrin and the two receptors TAS2R43 and TAS2R46.

**TABLE 2:** Concentrations of bitter sesquiterpene lactones in chicory, surrogate coffee and roasted coffee.

		Lactucin (1)	Lactucopicrin (2)	11β,13-Dihydrolactucin (3)
		μmol/g		
Fresh sample^a^	Radicchio	4.49 ± 0.51	0.88 ± 0.07	4.22 ± 0.07
	Chicory (spring 2021)	42.13 ± 4.10	11.93 ± 0.46	6.18 ± 0.19
Roasted sample^b^	Roasted coffee (100% Arabica)	n.d.	n.d.	n.d.
	Surrogate coffee (100% roasted chicory root)	32.48 ± 5.87	10.66 ± 0.94	52.69 ± 5.50
			μM	
Brews^c^	Roasted coffee (100% Arabica)	n.d.	n.d.	n.d.
	Surrogate coffee brew (100% roasted chicory root)	1.35 ± 0.10	0.45 ± 0.04	1.98 ± 0.05

Each sample was analyzed in triplicates.

^a^Concentration refers to fresh weight.

^b^Concentration refers to the solid, dry, ground material.

^c^Concentration refers to the beverage prepared by hot water percolation.

Roasted coffee: 48.75 g/750 ml water; Surrogate coffee: 30 g/750 ml water. n.d., not detected.

### Assessment of bitterness cross-adaptation between roasted coffee and surrogate coffee/chicory

**FIGURE 4:**
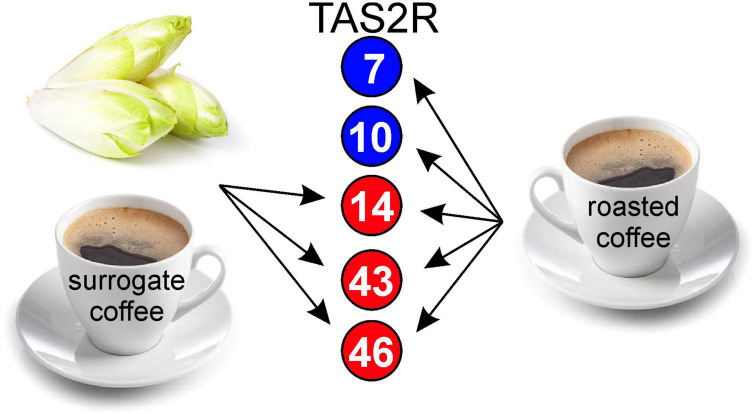
Schematic of TAS2R profiles activated by chicory/surrogate coffee and roasted coffee. Numbers in circles correspond to TAS2R subtypes. Red circles label receptors activated by chicory and surrogate coffee, blue circles label receptors exclusively activated by roasted coffee.

**FIGURE 5:**
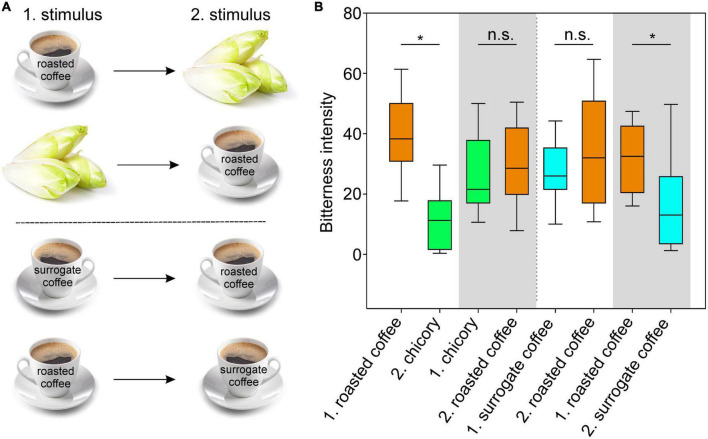
Sequential testing of roasted coffee, surrogate coffee and chicory. **(A)** Schematic outline of the sensory experiment. **(B)** Graph of the experimental results of sensory tests. Volunteers rated the bitterness of roasted coffee (brown boxes), fresh chicory leaves (green boxes), and surrogate coffee (blue boxes) in sequentially offered pairs. To allow for adaptation, the first stimulus (1) was kept 30 s in the mouth before expectorating. After a brief sip of bottled water, the second stimulus (2) was evaluated. Between the stimulus pairs (indicated at the *x*-axis, labeled by white/gray background colors), the experiment was interrupted for 30 min to allow recovery of bitter sensitivity. *Y*-axis, bitterness intensity. Statistically significant influences of the first stimuli on the second stimuli are indicated by asterisks *p*-values (*p < 0.02; n.s. = not significant). Statistics (One-way repeated measures ANOVA with Bonferroni’s *t*-test) were done using SigmaPlot (14.0).

The data obtained so far suggested that brews made from roasted coffee beans and surrogate coffee (chicory) exhibit very similar TAS2R activation profiles (Figure 4), which might have been the reason for using chicory as source plant for the generation of surrogate coffee. However, apart from the somewhat surprising overlap in the receptor activation profiles, we anticipated that this must have consequences for the perception of roasted coffee, surrogate coffee, and/or chicory when sequentially consumed. Therefore, we designed sensory experiments where volunteers were asked to sequentially taste pairs of samples and to rate the corresponding bitterness (Figure 5). The first pair of samples was chicory and roasted coffee. As evident in Figure 5B, the bitterness of chicory was rated lower after roasted coffee consumption. Evaluation of this stimulus pair in opposite sequence revealed no change in the bitterness of roasted coffee after chicory consumption. Note, that roasted coffee received in both settings similar bitterness ratings. This argues that roasted coffee with bitter compounds activating the 5 receptors, TAS2R7, −R10, −R14, −R43, and −R46 of which likely TAS2R43 and TAS2R46 dominantly contribute to the overall bitterness [cf. (13)], led to the substantial desensitization of the two receptors TAS2R43 and TAS2R46 activated by chicory bitter constituents (TAS2R14 should play no or only a minor role, cf. Figure 3). In contrast to that, if chicory was consumed first, at least two TAS2Rs, TAS2R7, and TAS2R10, would not desensitize affecting the overall bitterness of roasted coffee less. The next pair of samples tested was surrogate coffee and roasted coffee. Again, whereas the bitterness perception of roasted coffee was not significantly affected by the preceding gustatory evaluation of surrogate coffee, the bitterness of surrogate coffee was significantly reduced by previous consumption of roasted coffee. Therefore, we conclude that sequential consumption of beverages and/or food items that activate the same set of TAS2Rs can, depending of the sequence, strongly impact the taste profile of the stimulus tasted secondly.

## Discussion

### Identification of TAS2Rs responsive to chicory bitter substances

In the present study the TAS2R activation profile of bitter compounds from chicory and chicory-based surrogate coffee were analyzed and responses of three human bitter taste receptors, the TAS2R43, the TAS2R46 and, to a lesser degree, the TAS2R14, were observed. A previous publication investigating a similar set of four sesquiterpene lactones from edible *Asteraceae* species reported the activation of TAS2R46, thus confirming part of our study, however, activation of TAS2R14 and TAS2R43 was not observed (26). Whereas the previous study reported a threshold concentration of about 1 μM for lactucopicrin and an extrapolated EC_50_-concentration of 16.6 μM for the TAS2R46, our data demonstrated a 30–50-fold higher sensitivity for lactucopicrin for the same receptor. Since in our study the TAS2R46 showed the lowest threshold concentration for lactucopicrin and Yanagisawa and Misaka used this substance for the initial receptor screening, it seems reasonable to assume that sensitivity issues, perhaps related to the use of less sensitive receptor variants, prevented the identification of TAS2R43 and TAS2R14 in their study.

### Quantification of bitter compounds in chicory/surrogate coffee

Quantification of lactucin, lactucopicrin, and 11β,13-dihydrolactucin in fresh chicory, radicchio, and surrogate coffee powder as well as surrogate coffee brew (Table 2) demonstrated that, in particular the determined lactucopicrin levels fit well with the activating concentration ranges required for TAS2R43 and TAS2R46 (Figure 3 and Table 1). Hence, these two TAS2Rs are well suited to recognize the bitter compounds present in chicory in naturally occurring levels. Not surprisingly, roasted coffee did not contain detectable amounts of these bitter compounds (Table 2).

### Similarities in TAS2R activation profiles of roasted coffee and chicory/surrogate coffee

The activation pattern observed for the three chicory bitter substances are strikingly similar to those recently observed for novel bitter compounds identified in roasted coffee (13). In this study, the bitter substances mozambioside, bengalensol, cafestol and kahweol all activated TAS2R43 and TAS2R46 (13). Of the two receptors, the TAS2R43 was more sensitive and the maximal signal amplitudes elicited by the four compounds were in most cases higher than those observed with TAS2R46. Before this study, caffeine was shown to activate TAS2R7, −R10, −R14, −R43, and −R46 with rather low potencies (∼300 μM threshold conc.) (6).

Although the bitter compounds identified in roasted coffee and chicory are chemically diverse, the arrays of activated receptors are strikingly similar. As the use of chicory for the production of surrogate coffee was established long before the nature of bitter taste receptors has been discovered [in Germany production started in 1828 (27) but the recipe was supposedly inspired by already existing French recipes to cut roasted coffee], the question arises whether the choice for chicory as starting material for the production of surrogate coffee could have been based on the similarity in bitter taste. This touches an open question in taste research, namely whether it is possible to discriminate between different bitter compounds solely based on bitterness perception excluding confounding effects such as intensity differences, spatiotemporal differences or side tastes or smells [cf. (5) and references therein]. While some studies would suggest that this possibility exists (28–34), other studies argue against this possibility (35). The current study cannot solve this problem, however, it may allow to develop future sensory study designs including some of the here and in the previous coffee study investigated edible bitter compounds (13) as “undistinguishable” set combined with other bitter test compounds of different chemical classes.

### Cross-adaptation between roasted coffee and chicory/surrogate coffee

Another important finding of this study is the pronounced cross-adaptation between chicory/chicory-based surrogate coffee and roasted coffee. Whereas the tasting of roasted coffee as the first stimulus resulted in a significant reduction of the bitterness of chicory and surrogate coffee, the opposite sequence did not result in a similar reduction of the bitterness elicited by roasted coffee. This might be due to the only partial overlap of the TAS2R activation patterns, with chicory/surrogate coffee activating only three of the five TAS2Rs that respond to bitter compounds from roasted coffee (Figure 4). Nevertheless, one would anticipate that desensitization of 2–3 out of five TAS2Rs should already reduce the bitterness perception to a certain degree. This was not observed at a significant level. A possible explanation for this may come from another previous observation concerning the expression pattern of the 25 TAS2Rs in human circumvallate papillae (5). It was demonstrated that not all of the 25 receptors are co-expressed in bitter taste receptor cells (TRC). Currently, it is unknown if these subsets of bitter TRCs stochastically express distinct receptor sets or if more regulated gene selection events apply. The latter case could result in cell populations refractory to previous exposure to TAS2R14, TAS2R43, and TAS2R46 agonists and hence, allow full bitter signaling upon exposure to coffee-exclusive TAS2R7 and TAS2R10 agonists. As TAS2R43 and TAS2R46 belong to a primate-specific subset of TAS2Rs (3) and TAS2R14 is one of the closest relatives to this subfamily, a co-regulation and, as a consequence, co-expression in common subsets of bitter TRCs is conceivable (36, 37). TAS2R7 and TAS2R10 do not belong to this subfamily and may therefore mostly occur in other bitter TRC subsets.

## Conclusion

In our study, we have shown that chicory and a product made from chicory, surrogate coffee, exhibit a surprising overlap in bitter taste receptor activation profiles with roasted coffee. This overlapping receptor activation profiles resulted in substantial cross-adaptation between chicory/surrogate coffee and roasted coffee if consumed sequentially. This demonstrated that an exact knowledge of the receptor activation profiles of bitter compounds can be exploited to modify taste perception. While it is certainly not desired to provide a cup of coffee before a meal to increase the acceptance of children for bitter but healthy vegetables such as chicory, one could imagine to design tailored dishes before taking bitter medicine if receptor-matching can be achieved. There is an even wider way of thinking about our findings: In the past, rules orchestrating the perfect composition and sequence of food items consumed during a meal were coined. These rules are widely accepted, however, to date the molecular foundations for the observed taste and pleasure-raising effects are obscure. We have to anticipate that the sequence at which we eat our meals actually change the tastes of food items considerably and this may not be limited to bitterness perception. This is certainly an understudied field that should attract more research in the future.

## Data availability statement

The original contributions presented in this study are included in the article/Supplementary material, further inquiries can be directed to the corresponding author.

## Ethics statement

The studies involving human participants were reviewed and approved by the Ethical Committee of the Faculty of Medicine of the Technical University Munich (2022-203-S-NP). The patients/participants provided their written informed consent to participate in this study.

## Author contributions

MB: conceptualization. RL and TL: investigation. RL, TL, AD, and FZ: methodology. RL, TL, and MB: data curation. MB, RL, TL, and FZ: writing. MB, RL, TL, AD, and FZ: review and editing. All authors contributed to the article and approved the submitted version.
