# Supplementary material for: Overlapping activation pattern of bitter taste receptors affect sensory adaptation and food perception
Source: Front Nutr. 2022 Dec 19;9:1082698. doi: 10.3389/fnut.2022.1082698 (PMC9806268; doi:10.3389/fnut.2022.1082698)
Supplement: Supplementary file 1 [file Data_Sheet_1.docx]

Supplementary Material

Supporting table 1. Mass transitions, retention times, and calibrated range of the lactucin (1), lactucopicrin (2), and 11β,13-dihydrolactucin (3).

|  | Q1>Q3 | Rt. (min) | Calibrated linear  range (µM)^a^ | R^2^ | Precision (%)^a^ | Accuracy (%)^a^ |
| --- | --- | --- | --- | --- | --- | --- |
| Lactucin (1) | 276.97>213.0*/114.9 | 4.35±0.05 | 0.039 (LLoQ) – 5 | 0.998 | 1.1 – 8.1 | 96 – 107 |
| Lactucopicrin (2) | 411.04>215.0*/114.9 | 5.26±0.03 | 0.039 (LLoQ) – 5 | 0.995 | 1.7 – 8.5 | 93 – 107 |
| 11β,13-Dihydrolactucin (3) | 278.99>215.0*/159.0 | 4.10±0.06 | 0.039 (LLoQ) – 5 | 0.999 | 1.9 – 8.2 | 99 – 105 |
| ECHO standard (ES) | 276.98>213.0*/114.9 | 5.11±0.01 |  |  |  |  |
| ^a^ values of backcalculated standards, LLoQ Lower Limit of Quantification is defined as lowest concentration included into the calibration, precision <20% RSD, accuracy 80 – 120%. | | | | | | |


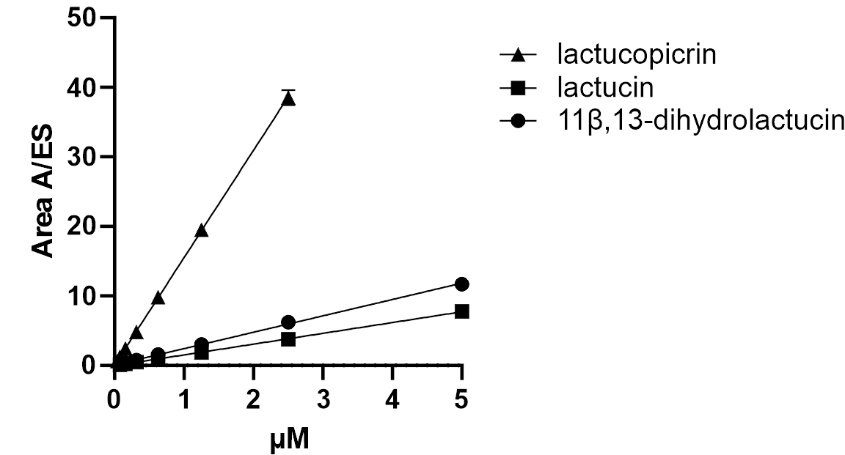


Supporting table 2. Quality controls. Recovery and precision of lactucin (1), lactucopicrin (2), and 11β,13-dihydrolactucin (3) in neat solution (10% ACN) and analyte-free matrix (coffee brew).

| Matrix |  |  | Lactucin | |  | Dihydrolactucin | |  | Lactucopicrin | |
| --- | --- | --- | --- | --- | --- | --- | --- | --- | --- | --- |
|  | Nominal  (µM) |  | Found  (µM, ±SD) | Accuracy,  Precision (%) |  | Found  (µM, ±SD) | Accuracy,  Precision (%) |  | Found  (µM, ±SD) | Accuracy,  Precision (%) |
| 10% ACN | 1.00 |  | 0.98±0.05 | 98%, 5.2% |  | 0.97±0.05 | 97%, 4.7% |  | 1.04±0.02 | 104%, 1.6% |
| Coffee | 1.00 |  | 0.91±0.02 | 91%, 1.9% |  | 0.84±0.03 | 84%, 2.7% |  | 0.86±0.04 | 86%, 2.7% |
| Data are means from n=3 | | | | | | | | | | |

| **No bitterness** | **Hardly perceived** | | **Weak** | **Moderate** | **Strong** | **Very strong** | **Strongest imaginable perception** |
| --- | --- | --- | --- | --- | --- | --- | --- |
|  |  |  |  |  |  |  |  |
| **example** | 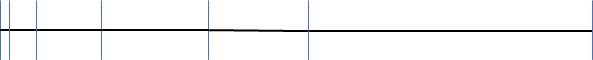 | | | | | | |

**Strong, with the tendency „very strong“**

Supporting figure 1. Scale for sensory evaluation of perceived bitterness.
